# A closer look at sex pheromone autodetection in the Oriental fruit moth

**DOI:** 10.1038/s41598-022-10954-x

**Published:** 2022-04-29

**Authors:** Alicia Pérez-Aparicio, Byrappa Ammagarahalli, César Gemeno

**Affiliations:** 1 Department of Crop and Forest Sciences, University of Lleida, Av. Alcalde Rovira Roure 191, 25198 Lleida, Spain; 2 Department of Crop and Forest Sciences, University of Lleida-Agrotecnio-CERCA Center, Av. Alcalde Rovira Roure 191, 25198 Lleida, Spain; 3 Present Address: Gaiagen Technologies Pvt Ltd (Formerly Pest Control India Pvt Ltd), Bengaluru, 562163 India

**Keywords:** Olfactory receptors, Entomology

## Abstract

Female moths emit sex pheromone to attracts males, and although they are not attracted to their own sex pheromone, they appear to detect it as it affects their behavior. In order to elucidate the mechanism of pheromone “autodetection” we compared responses of olfactory receptor neurons (ORNs) of male and female *Grapholita molesta*, a species with reported pheromone autodetection. Two concentrations of the major (*Z*8-12:Ac) and minor (*E*8-12:Ac) sex pheromone components, a plant-volatile blend containing methyl salicylate, terpinyl acetate and (*E*)-β-farnesene, and the male-produced hair-pencil (i.e., courtship) pheromone (ethyl trans-cinnamate) were tested in 45 male and 305 female ORNs. Hierarchical cluster analysis showed radically different peripheral olfactory systems between sexes that could be linked to their specific roles. In males 63% of the ORNs were tuned specifically to the major or minor female sex pheromone components, and 4% to the plant volatile blend, while the remaining 33% showed unspecific responses to the stimulus panel. In females 3% of the ORNs were specifically tuned to the male hair-pencil pheromone, 6% to the plant volatile blend, 91% were unspecific, and no ORN was tuned their own sex pheromone components. The lack of sex pheromone-specific ORNs in females suggests that they are not able to discriminate pheromone blends, and thus pheromone autodetection is unlikely in this species. We discuss our results in the context of the methodological limitations inherent to odor stimulation studies.

## Introduction

Female moths produce a mixture of related fatty acid derivatives that is liberated from their protruded abdomen tip during the calling period and serves as an attractant to conspecific males. Although some species use only one pheromone component to communicate, they generally synthetize a more complex blend that includes major and minor behavior inducing compounds that, in the proper amount, can increase the attraction of males^1^. Moth sex pheromones are of variable nature, and slight differences in carbon chain length, double bond location, functional group and blend composition favor discrimination not only between species, but also among populations or strains of the same species^1^. The European corn borer (ECB) *Ostrinia nubilalis* Hübner, for instance, exists as two separate sex pheromone races. ECB(Z) females produce a 97:3 blend of *Z*11- and *E*11- tetradecenyl acetate, whereas ECB(E) females produce the opposite 1:99 ratio of the Z and E isomers. Males of each race respond specifically to the blend produced by females of their respective race^2,3^.

We are therefore presented with a highly specific communication system, where the qualitative nature of the compounds is entwined with a quantitative proportion of each of the components of the pheromone blend. Male moths rely on the precise species-specific blend to locate and fly towards potential mates from relatively large distances, and thus they have evolved an olfactory system that detects pheromone components with great sensitivity and specificity over a wide range of concentrations^4,5^. Males present long trichoid sensilla along the antennae that house different olfactory receptor neurons (ORNs) types, each specifically tuned to each one of the components of the female pheromone blend^5,6^. ORNs are generally tuned to one or a few biologically significant compounds to which they respond strongly^7^, and pheromone receptor neurons (PRNs) stand at the high end of the specificity spectrum^5^. The composition of a pheromone blend is represented across the existing types of specialist PRNs on the antennae^2^, which distribution follows two distinct patterns in males. In many species each pheromone receptor neuron is housed in a different sensillum trichodeum while in other species different PRN types, usually just two, share the same sensillum^4^. These two arrangements appear to confer males with an efficient mechanism to continuously track the chemical identity of the pheromone plume along the widely variable concentration range they experience when flying upwind towards females^4^. Axons of ORNs arborize in the antennal lobe, where glomeruli organize in relation to function, a bigger glomerulus indicating a higher number of ORN inputs, or larger dendritic diameters. In males, a specific structure called the macro-glomerular complex contains a large glomerulus that receives the input of the ORNs tuned to major-pheromone-component^2^.

Although females are not known to orient to their own sex pheromone like males do, there is increasing evidence that they do respond to conspecific sex pheromone with changes in reproductive behaviors such as pheromone emission, oviposition and mating^8^. Holdcraft et al.^8^ define “female autodetection” as when “females can detect their own sex pheromone”. To the extent that pheromone “autodetection” implies spotting the presence of conspecific females in the area (in order to maximize reproductive fitness by changing pheromone emission behavior^8^), the definition of autodetection should also imply that females are able to discriminate their conspecific blend from similar ones released by closely related species. In order to distinguish pheromone blends, females should possess an olfactory system akin to males that, at the very least, is able to detect each pheromone component independently. While the mechanism of pheromone detection of males is well known, much less is known about sex pheromone detection in females, specifically at the ORN level^8^. The scarce single-sensillum recording (SSR) studies on females indicate that, with few exceptions, they lack true PRNs^8^. This would account for the absence of a MGC in female brains and for the smaller size of anatomically analogous glomeruli in its place^2^.

The aim of our study is to determine if female moths are equipped with ORNs similar to those of males to detect their own sex pheromone. To address this question we used the Oriental fruit moth, *Grapholita molesta* Busck. This species is a major pest of peaches and apples worldwide, and it is successfully controlled by mating disruption so its pheromone communication system has been thoroughly studied^9–11^. A very precise ratio of the major, *cis*-8-dodecenyl acetate (*Z*8-12:Ac), and minor, *trans*-8-dodecenyl acetate (*E*8-12:Ac), pheromone components (approx. 100:6) is necessary to achieve optimal attraction^10,11^. In males 64% and 7% of the olfactory receptor neurons housed in separate sensilla trichodea respond specifically to *Z*8-12:Ac and *E*8-12:Ac, respectively^12^, these numbers being roughly proportional to the ratio of the acetate compounds in the female pheromone blend^13^. The remaining 29% of the male sensilla do not respond to pheromone components, but some respond to plant volatiles^14^. Accordingly, males have a macro-glomerular complex in the antennal lobe, whereas the equivalent antennal lobe location of females presents glomeruli of ordinary size^15^.

Alterations in the calling period of *G. molesta* females previously exposed to conspecific pheromone, diminished reproductive success under similar conditions, and EAG responses to the pheromone in female antennae supports pheromone autodetection in this species^8^. However, evidence of pheromone autodetection at the ORN level is missing. In this study, we obtained SSR from male and female antenna of *G. molesta* using the two main female sex pheromone components (*Z*8-12:Ac and *E*8-12:Ac). In addition, we tested the plant volatiles terpinyl acetate, (*E)*-β- farnesene, and methyl salicylate, which are behaviorally active and stimulate male ORNs^14,16,17^. *G. molesta* males produce a courtship pheromone from their everted hair-pencils that is fundamental for females to accept mating and we tested its main compound, ethyl *trans*-cinnamate^18^, as we expected that females may have specialized ORNs to detect it. In order to facilitate the location of sensilla for SSR we mapped their location along the antenna of females using SEM, as reported for males^12^.

## Results

### Morphology

**Figure 1:**
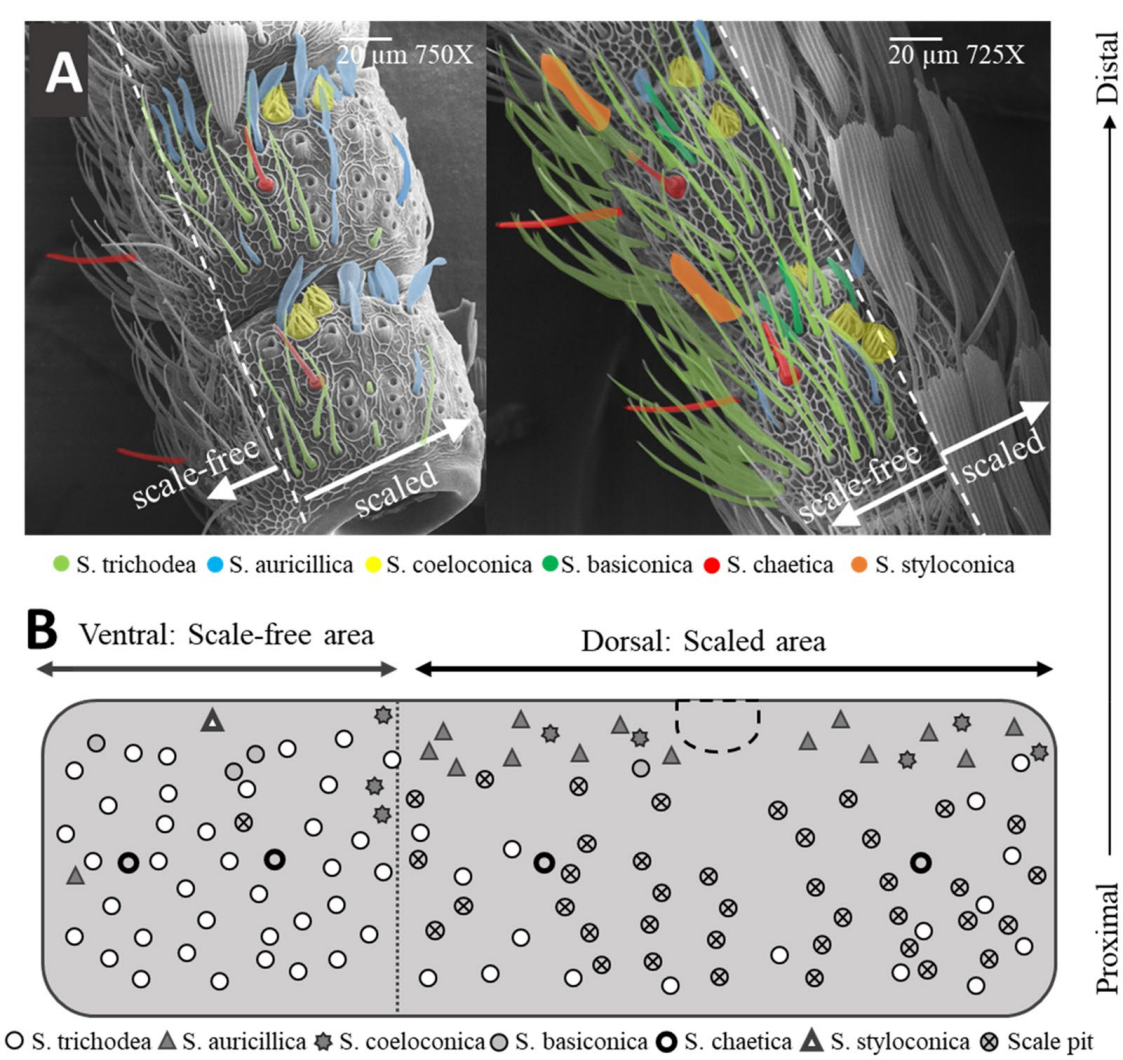
Distribution of sensilla types on the antenna of *G. molesta* females. (**A**) Different sensilla types are shown with different colors in the SEM picture of flagellomeres 25th and 26th. (**B**) Distribution of sensilla and scales on a representative flagellomere located towards the middle of the antenna.

SEM revealed 6 types of sensilla (Fig. 1). In all flagellomeres 4 sensilla chaetica can be found, and in the scale-free region it is frequent to encounter 1 sensillum styloconicum on each flagellomere. The most abundant sensilla type is sensilla trichoidea (54%), followed by auricillica (27%), coeloconica (8%) and basiconica (4%) (Supplementary Table S1 online, and Supplementary Fig. S2 online).

### Dose–response curves

**Figure 2:**
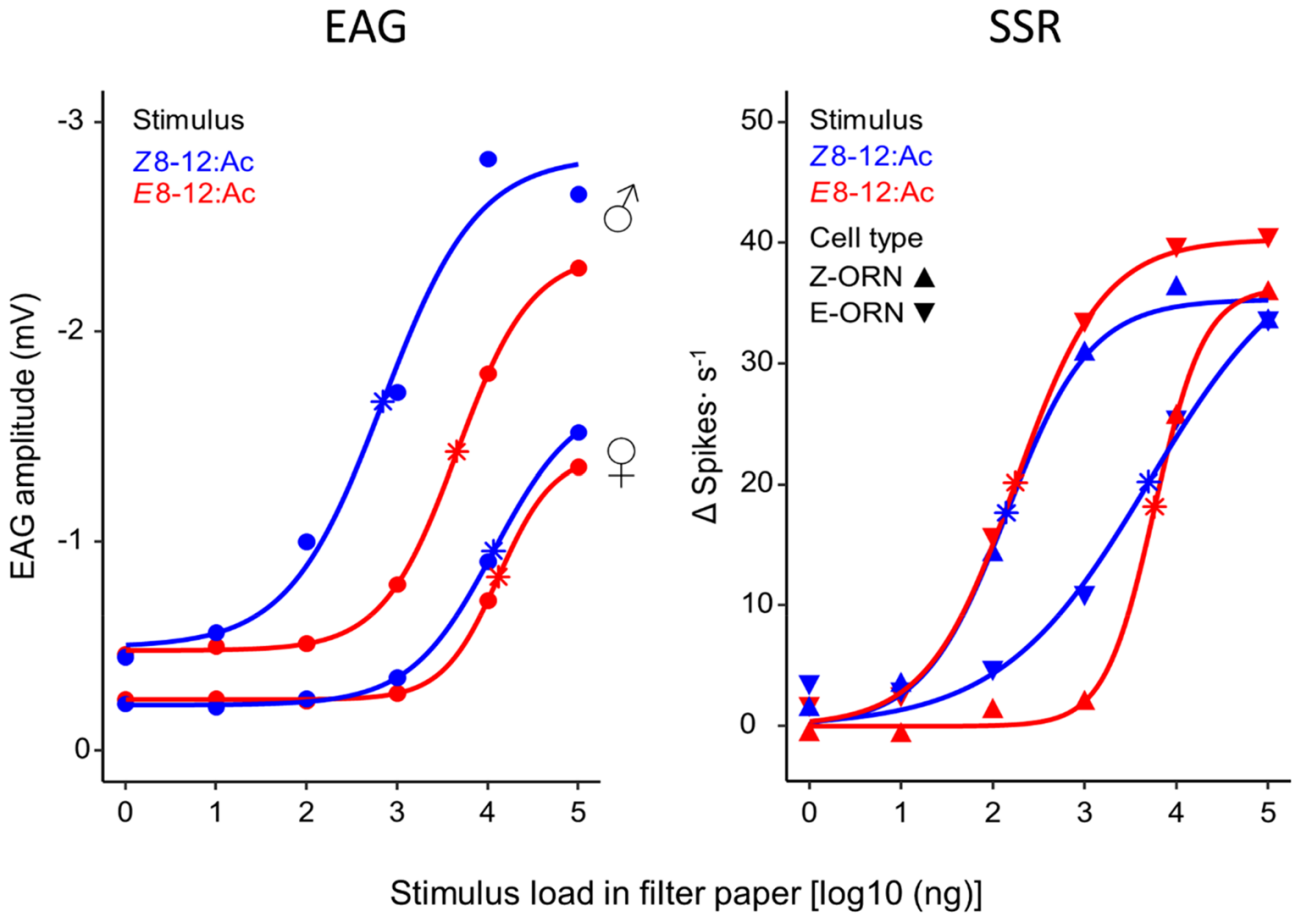
Electrophysiological dose–response curves of *G. molesta* to the major (*Z*8-12:Ac) and minor (*E*8-12:Ac) pheromone components. Left: EAG of male and female antennae. Right: SSR of male Z- and E-ORNs. Symbols are average response and curves are the predicted values from fitted logistic functions. Asterisks indicate estimated dose-50 (ED_50_).

Antenna and ORN sensitivities were assessed using the ED_50_ estimated from the dose–response curves (Fig. 2, Table 1). The antenna of males was more sensitive than the antenna of females to both sex-pheromone components. The antenna of males was more sensitive to the Z-isomer than to the E-isomer, whereas the antenna of females did not discriminate between them (Table 1, Fig. 2; means with errors are shown in Supplementary Table S2 online, and responses from individual antennae are shown in Supplementary Fig. S3 online). At the ORN level, Z- and E-ORNs were more sensitive to their respective isomers than to the other one. Furthermore, Z- and E-ORNs were equally sensitive to their respective isomers, and equally insensitive to the other isomers (Table 1, Fig. 2, means with errors are shown in Supplementary Table S3 online, and responses from individual ORNs are shown in Supplementary Fig. S4 online).

**Table 1:** Estimated dose-50 (ED_50_) of *G. molesta* EAG and SSR dose–response curves.

**EAG**		
Antenna	Stimulus	ED_50_ (mean ± SEM, ng)
Male	*Z*8-12:Ac	495.30 ± 190.65a
	*E*8-12:Ac	4174.50 ± 1665.20b
Female	*Z*8-12:Ac	10,545.70 ± 3488.30c
	*E*8-12:Ac	20,726.80 ± 8236.80c

**SSR**		
ORN type	Stimulus	ED_50_ (mean ± SEM, ng)
Z-ORN	*Z*8-12:Ac	144.00 ± 44.78a
	*E*8-12:Ac	5616.77 ± 1582.15b
E-ORN	*Z*8-12:Ac	4931.43 ± 2315.70b
	*E*8-12:Ac	175.40 ± 62.59a

Different letters indicate significant differences among the 4 EAG curves and among the 4 SSR curves (ANOVA, *P* < 0.05).

Based on the results of the dose–response curves, we chose the 10^2^ and 10^4^ ng concentrations to characterize ORN types (see below). With the low dose (10^2^ ng) Z- and E-ORNs could be discriminated in males, but the female antenna was insensitive to this concentration. With the high dose (10^4^ ng) the female antenna started to show responses, indicating some ORN activity, and the male Z- and E-ORNs could still be separated from each other, despite being close to saturation.

### ORN classification

**Figure 3:**
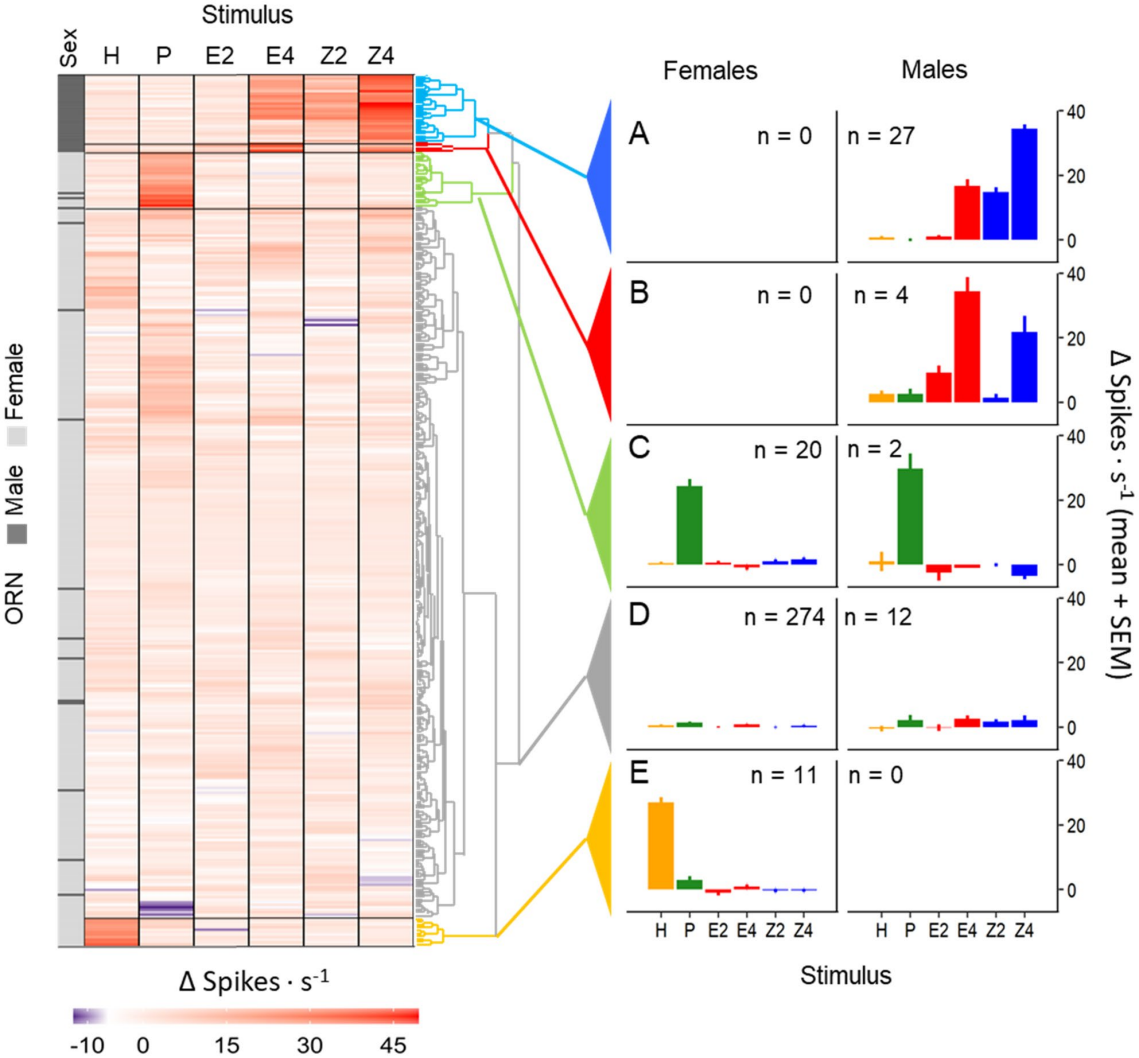
Classification of ORN types. The left panel shows a hierarchical cluster analysis (HCA) grouping *G. molesta* female and male ORNs (n = 305 and 45, respectively) according to their responses to 6 odorant stimuli [H: male courtship pheromone (i.e., hair-pencil pheromone) at 10 µg; P: plant blend at 100 µg; E: minor pheromone component *E*8-12:Ac; Z: major pheromone component *Z*8-12:Ac, each at 2 concentrations (100 ng = 2) and (10 µg = 4)]. Each entry in the y-axis indicates a different ORN color-coded by sex (grey for female, black for male). The horizontal lines separate HCA clusters which have been color-coded in the dendrogram on the right side and represent 5 physiological ORN types. The right panel shows average responses for each of the 5 HCA clusters: (**A**) Major sex pheromone component ORNs, (**B**) minor sex pheromone component ORNs, (**C**) Plant-responding ORNs, (**D**) ORNs with unspecific responses to the stimulus panel, and (**E**) Male hair-pencil pheromone ORNs. The number of ORNs for each physiological type is indicated (“n”).

Single sensillum recordings were made on 239 and 42 sensilla trichodea from a single antenna of 50 females and 12 males, respectively. Most male ORNs exhibited background firing rates of between 0 and 10 spikes·s^−1^, while the distribution of spontaneous activity in female cells was broader, between 10 and 20 spikes·s^−1^ (Supplementary Fig. S5 online). Most female sensilla contained 1 to 3 active ORNs per sensillum, while in males only 3 recordings presented more than one cell. Hierarchical cluster analysis grouped the 305 female and 45 male ORNs in 5 distinct clusters showing specific response patterns to the selected stimuli (Fig. 3). In females, the largest group comprised the so called “unspecific” ORNs (90%) (Fig. 3). In these neurons responses were weak in general and none of the test stimuli was clearly better than the other ones, and thus they were unspecific to the panel of test odors. Unspecific ORNs comprised 27% in males. A second group of ORNs responded strongly to the plant blend and comprised 6.5% of the total in females and 4% in males. A third group that responded specifically to the hair-pencil male pheromone (ethyl *trans*-cinnamate) contained 3.6% of the female cells and no male cells (Fig. 3). The last two groups of cells responded strongly and preferentially to either the major or the minor female sex pheromone components. The Z-ORN group contained 54% of the male ORNs and the E-ORN group contained 8% of the male ORNs. None of the 305 female ORNs belonged to the Z- or E-ORN groups according to the HCA (Fig. 3).

## Discussion

In order to discriminate odor blends having slightly different ratios of similar odorants it is crucial that receivers bear sensory cells specifically tuned to each one of the components of the blends. Male moths have pheromone receptor neurons (PRNs) specifically tuned to each one of the two or three compounds that typically comprise the species-specific sex pheromone blend released by conspecific females^19^. If females had to detect their own sex pheromone and discriminate it from similar ones, they would presumably require a pheromone detection system similar to that of males. However, males use the sex pheromone to locate females, whereas females presumably use it to detect the presence of other females^8^, so the pheromone detection system of males and females may not need to be alike. We tested this hypothesis in the Oriental fruit moth *G. molesta* by means of electroantennography (EAG) and single sensillum recordings (SSR).

At the EAG level, which represents the combined activity of ORN receptor potentials across the antenna^5,20,21^, we found that males were approximately 20 and 5 times more sensitive than females to the major (*Z*8-12:Ac) and minor (*E*8-12:Ac) pheromone components, respectively. This indicated that either the female antenna was equipped with a lower proportion of pheromone-responding cells than males, that these cells were not as sensitive as those of males, or both. Furthermore, the male antenna was about tenfold more sensitive to *Z*8-12:Ac than to *E*8-12:Ac, whereas the female antenna had similar sensitivity to the two isomers, suggesting additional sex differences in pheromone detection.

In order to uncover the cellular mechanisms behind sex differences in pheromone perception, we tested the response of male and female ORNs to the sex pheromone, plant volatiles and the male-produced hair-pencil pheromone. Hierarchical cluster analysis grouped ORNs in 5 neatly separated response classes. ORN responses agreed with the EAGs because in males most of the cells sampled were major-component sex pheromone specialists, and correspondingly the male EAG was about tenfold more sensitive to the major than to the minor pheromone component. In contrast, female ORNs responded very weakly and unspecifically to sex pheromone, and thus EAG responses of female antennae to sex pheromone were very small and did not discriminate between the two sex pheromone isomers. Thus, the smaller EAG response of female *G. molesta* to sex pheromone is not due to a smaller number of male-equivalent PRN than in males, but to the summed response of many unspecific ORNs with weak pheromone responses.

ORN responses to sex pheromone have been reported in females of several moth species^8^. Two of them, *Spodoptera littoralis* (Boisduval) and *Heliothis virescens* (F.), have been studied in substantial detail. *Spodoptera littoralis* have long and short sensilla trichodea, but while the former occur abundantly throughout the male flagellomere (80 in each one), in females they are located exclusively on the lateral edges of the flagellomere, six on each side^22^. In males about 80% (in a sample of 125 sensilla) of the long sensilla trichodea house a single ORN that responds specifically to the major pheromone component (*Z*9,*E*11-14:Ac), and the remaining 20% respond to minor pheromone components^22^. In females 52–98% (in samples of 40–120 sensilla) of their few long sensilla trichodea house one cell that responds mainly to the major pheromone component, and dose–response curves show that it is as sensitive as the male PRNs^22,23^. Molecular analysis supports physiological observations, showing a 50-fold higher expression receptor protein of the major pheromone component SlitOR5 in male than in female antennae^24^, and also higher expression of receptor genes for minor pheromone components in male than in female antennae^24,25^. By comparison, *H. virescens* males have many more long than short sensilla trichodea, and females only bear the short type^26^. The majority of the long trichoid sensilla of males (80% in a sample of 403) house a neuron that responds specifically to the major pheromone component (*Z*11-16:Ald), whereas 3% of the sampled neurons respond specifically to the minor component (*Z*9-16:Ald). The remaining 16% respond to heterospecific pheromone compounds with somewhat lower specificity than the PRNs^27–29^. A small percentage of male ORNs in short sensilla trichodea (15% in a sample of 202) are major component specialists, and 38% respond to minor pheromone components with diverse specificity^30^. In females, however, only 2% of the cells out of 184 recordings from the short sensilla trichodea responded to the major compound and 37% of the cells responded to the minor compound or heterospecific sex pheromone components, and in both cases the responses were weak and not too specific^31^. Corresponding with the physiological data of *H. virescens* the expression of the olfactory receptor genes HvirOR13 and HvirOR6 associated with sex pheromone components is significantly larger in male than in female antennae^32^.

Other moth species have been studied in less detail. A sample of 75 female *Agrotis segetum* (Denis & Schiffermüller) ORNs showed no pheromone responses^33^, and in *Manduca sexta* (L) similar pheromone ORN dose–response curves were observed in both sexes, but it was to a minor component and only 8 cells out of 200 were of this type^34^. Weak response to the major pheromone component 9*Z*,12*E*-14:Ac in one out of 36 female neurons of *Cactoblastis cactorum* (Berg) were recorded^35^, and five *Trichoplusia ni* (Hübner) female sensilla harboring a neuron with similar threshold responses to the major pheromone component as male neurons have been reported, but the number of cells sampled was relatively small^36^.

Altogether, *S. littoralis* (and perhaps *T. ni*) emerge as species where females appear to have true PRN equivalent to those of males, while in the other species studied, including *G. molesta*, the evidence of PRN in females is scarce or lacking, and in every case pheromone-responding ORNs occur in much smaller numbers in females than in males. It could be argued that females do not need as many, or as sensitive, PRNs as males to detect the presence of other females. However, females do not appear to have PRNs specifically tuned to each ingredient of the pheromone blend, and thus they should not be able to discriminate their own pheromone blend from similar ones, as males do. For example, *S. littoralis* females have an ORN type which sensitivity, as per dose–response curves, is equivalent to that of the major component PRN of males^22^, but ORNs responding to the minor components, *Z*9-14:Ac and *E*11-14:Ac, have not been detected in females^37^. Furthermore, female moths lack the behavioral antagonist ORNs that males of some species have in order to prevent cross-attraction between species that produce very similar pheromone blends^38^. Thus, the overall evidence available today indicates that although females of some moth species appear to have major component PRNs, no females are endowed with ORNs tuned individually to both, the major and minor pheromone components, or the pheromone antagonists, and therefore females, unlike males, are probably unable to discriminate their own pheromone from those of related species. So, how can we explain the substantial evidence of pheromone autodetection in female moths^8^?

We believe that to unambiguously establish pheromone autodetection it should be first demonstrated that females are able to behaviorally discriminate their own sex pheromone blend from similar ones, otherwise they may just be detecting pheromone components irrespective of their taxonomic relationship. This procedure is standard in sex pheromone identification where males are presented with different blend ratios and often prefer the one of conspecific females^1^, but as far as we know this approach has not been applied yet in pheromone autodetection tests^8^. Secondly, many olfactory autodetection tests employ unnaturally high pheromone doses for prolonged periods of time, which may result in altered odor perception due to sensory adaptation or stimulation of non-target receptors. For instance, the behavioral tests that demonstrated pheromone autodetection in *G. molesta* employed 10–100 µg pheromone loads on rubber septa for up to 10 h on caged females^39–41^. These experimental conditions were chosen to emulate the environment that theses insects experience under mating disruption in the field. Because EAG responses (and the number of matings) were reduced 24 h after exposure^39–41^, it is very likely that females experienced acute sensory adaptation and so their capacity to sense or discriminate sex pheromone was probably altered.

Most ORN dose–response curves typically show that, at natural concentration ranges, a given odorant stimulates a narrow spectrum of receptor neuron types, but at abnormally high concentrations additional receptor types (that would not typically respond to that odorant at natural doses) may also be excited^7,42^. The displacement of the dose–response curve along the concentration axis represents the affinity between odorant and receptor (estimated with the ED_50_), while the height of the curve (i.e., the firing rate) indicates the maximum number of ORs activated in that ORN^43^. A cell that is very specific for a given ligand will have a very low ED_50_ to it relative to other ligands, independently of the firing rate. For example, the dose–response curves of male *G. molesta* PRNs show that the ED_50_ of the Z- and E-cells is lower to their respective ligands than to the other one, but as the curves approach saturation the cells fire at similar rates and thus become less specific at the highest doses (Fig. 2 and^12^). The same occurs in ORNs tuned to non-pheromone stimuli^7^, so it is possible that autodetection experiments that use unnaturally high pheromone doses may stimulate non-pheromone neurons (like the unspecific neurons of *G. molesta* that we describe in here). This unspecific activation could falsely signal the brain the presence of non-pheromone odors which could then trigger behavioral responses that the sex pheromone at natural concentrations would never do.

Another very important (but often neglected) confounding factor in odor sensation studies is the presence of impurities in the sample, which is exacerbated at very high stimulus concentrations. This has been substantially demonstrated in a study which explored the unlikely response of a food volatile-sensitive ORN of *Drosophila melanogaster* to the sex pheromone of female silk moths, *Bombyx mori*^44^. Careful chemical analyses and the use of GC coupled to SSR (GC-SSR) demonstrated that a minute (< 1%) content of plant volatiles in the moth pheromone sample was responsible of the unexpected response of *D. melanogaster* ORNs. This study shows that chemicals that are not detected by GC can be hugely magnified in the volatile sample, particularly if they are more volatile than the test stimulus they evaporate with. It also highlights the importance of using GC-SSR as perhaps the only way to stablish a true causal relationship between volatile stimulus and response. The impact of impurities in olfactory behavior studies is probably even greater than in electrophysiological studies due to the larger sensitivity of the former. In our study of *G. molesta* the practical absence of SSR responses of one sex to the sex pheromone of the opposite sex, and the high differences in the ED50´s of male and female EAGs suggests that the effect of the inevitable impurities of the samples is not too large. But a GC-SSR study would be required to confirm this point.

Calling females would be a more suitable source of pheromone stimulus in autodetection experiments than the synthetic blends, since they release a natural concentration of the optimal blend. Indeed, the calling period of target females is affected by neighboring calling females^45–47^. This change in behavior is very reasonable evidence of pheromone autodetection. Yet, as with synthetic stimuli, if conspecific and heterospecific calling females are not compared alongside then there is no definitive demonstration that females can discriminate their own sex pheromone from similar ones, and thus pheromone autodetection cannot be demonstrated. Using calling females requires that other sensory modalities (e.g., visual, acoustic) are controlled for.

Our study shows that *G. molesta* females have specific ORNs to stimuli other than their own sex pheromone. Testing compounds other than the sex pheromone in an adequate number of cells in both males and females was decisive in separating cell types with cluster analysis. Testing male and female ORNs together was also crucial considering the notorious methodological variation across olfactory setups^48^, and it also allowed direct comparison of the established response of male ORNs to sex pheromone with the poorly characterized response of female ORNs. The clear separation of ORNs by stimulus type in both sexes indicates that the absence of sex-pheromone specific cells in females did not result from an inadequate sample size or faulty test stimuli. In male *G. molesta* 29% of the sensilla trichodea do not respond to pheromone^12^, which is not far from the 38% that we report here. The presence of plant-volatile sensitive ORNs in males is believed to increase their probability of finding females, since these odorants have a synergic effect on male response to pheromone^14,16,17,49,50^. The low number of plant ORNs found in females probably resulted from testing only 3 plant compounds. Testing further ecologically relevant compounds will probably increase the number of cells responding to host odorants^14,16^. Females also have a higher number of auricilic sensilla than males^14^, and recordings from these sensilla would probably increase the number of plant responding ORNs in *G. molesta* males and females^51^.

Our SSR experiment further revealed that 3% of the female sensilla are specifically tuned to ethyl trans-cinnamate, a close-range volatile that males release during courtship^18^. In the noctuid moth *H. virescens* 33% out of 184 female ORNs responded to the male hair-pencil pheromone and to an interspecific behavioral antagonist^31^. Interestingly, ORNs of a similar physiological type were also reported in males of *H. virescens* (19% out of 202 cells)^30,52^. Conversely, we found no ORNs tuned to the male hair-pencil pheromone, ethyl *trans*-cinnamate, in male *G. molesta*. Male *G. molesta* produce additional hair-pencil pheromone components^18^ that may disclose additional ORN types when tested.

Our study shows that the organization of the olfactory sensory system of male and female *G. molesta* appears to be shaped by the specific function of each sex. Females and males seem to be relatively unable to detect their own pheromones but have ORNs that respond to the pheromone of the opposite sex and to ecologically relevant plant stimuli. Although PRNs have been described in females of other species, close scrutiny reveals that their physiological and biological relevance appears to be overrated. However, there is field evidence that female behavior can be altered under mating disruption conditions where the environmental level of pheromone surpasses the natural concentration that they themselves release^45^. There are also indications that mating disruption delays the age at which females mate and this could reduce their reproductive output^53^. Females could change their behavior even without detecting the pheromone if, for example, they leave the area if males are not attracted to them. Further studies on the effect of sex pheromone on female behavior are wanted to establish the effect of mating disruption on females, even if this effect is not mediated by pheromone autodetection *sensu stricto*.

## Materials and methods

### Insects

The colony of *G. molesta* was established with insects collected in peach orchards in Piacenza, Italy, and has been maintained at the University of Lleida, Spain, since 2005. Larvae were reared on semisynthetic modified diet^54^ under a L16:D8 photoperiod at 23 ± 1 °C. Male and female pupae were placed in separate environmentally controlled chambers inside 1L polypropylene containers provided with 10% sucrose solution drinkers. Adults were collected every 2–3 days after hatching and used when 2–4 days old.

### Scanning electron microscopy

The number and distribution of sensilla types on the female antennae followed the same SEM procedure of the male antennae^12^. Briefly, antennae were dissected from live individuals and the scales were removed mechanically. The antennae were mounted on SEM stubs lined with conductive tape and dried at room temperature before gold sputter coating. The scale-free (ventral) area of 8 antennae and the scaled (dorsal) area of 5 antennae, each from a different individual, were examined. Sensilla counts were made every 5th flagellomere, starting on the proximal one. Total sensilla count per antennae was estimated by extrapolating these counts to the skipped flagellomeres. The scale-free area, which covers one third of the perimeter of each flagellomere, was fully visible in all the samples but the scaled area, which covers the remaining of the flagellomere, was always partially obstructed from vision due to the cylindrical shape of the antenna. Using characteristic landmark structures that indicated the sagittal axis on the scaled area we could extrapolate sensilla counts from the visible section of the scaled area to the section hidden from view. Abundance and pattern of distribution of all types of sensilla are reported.

### Odorants and stimulation

The sex-pheromone components *Z*8-12:Ac (CAS 28079-04-1) and *E*8-12:Ac (CAS 40642-40-8) were provided by Pherobank (Wijk bij Duurstede, The Netherlands) with an initial purity of 99%. GC-MSD analysis revealed a Z:E isomer content of 100:0.301 in *Z*8-12:Ac and of 0.633:100 in *E*8-12:Ac. The male produced hair-pencil pheromone, ethyl *trans*-cinnamate (CAS 18794–84-8, Fluka product number 96350, lot and filling numbers 1105301 and 13407214, respectively, ≥ 98%), and the plant odors (*E)*-β-farnesene (“FAR”, CAS 18794-84-8, Sigma Aldrich product number 73492, > 90% pure), methyl salicylate (“MS”, CAS 119-36-8, a present from Ashraf El-Sayed, New Zealand), and terpinyl acetate (“TA”, CAS 80-26-2, Sigma-Aldrich product number W20470-0-K, lot number 06703D407, ≥ 95% pure), did not show significant contamination peaks by GC-MSD.

Chemicals were loaded in 10-*µ*l aliquots onto *n*-hexane pre-cleaned and folded over filter paper pieces (0.5 × 1 cm, Whatman #1, Sigma-Aldrich, Spain). After solvent evaporation (5 min), they were introduced into 1-ml disposable plastic pipettes (73-mm long × 7-mm i.d. at the wide end, 1-mm i.d. exit hole). A 40-mm section of silicone plastic tubing (5-mm i.d. and 8-mm o.d., Sigma-Aldrich product number BR143358) was inserted into the larger opening of the pipette tip and the odor cartridges were stored in glass test tubes with PTFE-lined screw caps to be used within the day. A given cartridge was not used for more than 20 stimulations. Filter papers formulated with solvent (*n*-hexane) controlled for puffing effects in the EAG test. The test tubes that kept the stimulus cartridges were rinsed with acetone and heated at 250 °C overnight before being reused. A 0.5 l/min charcoal-filtered and humidified air flow blew continuously over the insect preparation through an 8-mm i.d. PTFE tube placed 15–20 mm from the preparation (air velocity at exit = 0.16 m/sec). The tip of the odor cartridge bearing the filter paper was positioned 8 cm upwind from the insect and perpendicular to the direction of the continuous air flow. Charcoal-filtered room air was puffed at 0.2 l/m through the odor cartridge to the continuous flow tube for 200 ms. Time interval between puffs was at least 30 s, but longer if needed to let the spike activity return to pre-stimulation levels. A maximum of 10 cells were recorded per insect, and at least 5 min elapsed between recordings from two different cells. The air around the preparation was constantly exhausted to minimize contamination.

### Electrophysiology

Moths were immobilized with CO_2_ and held in an aluminum block. The protruding head was restrained with tape and the antennae either remained free (for EAG) or were fixed to double-sided tape using minute strips of smoking paper (for SSR). Tungsten wire electrodes (0.125-mm diameter, 99.98% purity, Advent Research Materials Ltd, England) were electrolytically sharpened with 10% KOH. Pulled glass-capillary electrodes were filled with physiological saline solution (1% NaCl in distilled water) and housed 0.5 mm diameter platinum wires. A glass electrode inserted through the mouthparts and connected to ground served as the reference electrode. For EAG the recording glass electrode contacted the cut tip of one antenna. For SSR, the recording tungsten electrode was placed near the base of a randomly chosen sensillum trichodeum and pushed gently inward with the help of a manual micromanipulator (NMN-25, Narishige, Japan) until action potentials were observed.

Sensilla were randomly sampled along the antennae, including the scaled region after removing the scales with the help of a tungsten electrode. The signal from the recording electrode was pre-amplified (10 × gain), further amplified and band-pass filtered at 0.1 to 3 kHz for SSR and 0.1–100 Hz for EAG (Model 300 AC/DC Differential Amplifier, AM-Systems, Sequim, Washington, USA), and digitized (MICRO4 CED 1401, Cambridge Electronic Design Limited (CED), Cambridge, England). Spikes were sorted with Spike2 (CED).

### Dose–response curves

Dose–response curves for both EAG and SSR used six concentrations of *E*8-12:Ac and *Z*8-12:Ac (1–10^5^ ng). The order of the stimuli was first the negative control (*n*-hexane, for EAG), followed by low to high doses of *E*8-12:Ac, and then low to high doses of *Z*8-12:Ac. For SSR an initial puff of a 1:1 blend of *Z*8-12:Ac and *E*8-12:Ac (10^3^ ng of each) determined whether the cell was pheromone-responsive. Responding cells were then stimulated with a high dose of *E*8-12:Ac (10^3^ ng) to determine if they were of the *Z*8-12:Ac type or of the much less abundant *E*8-12:Ac type^12^ before proceeding with the dose treatments. For EAG, 21 male and 20 female antennae were tested and for SSR 6 Z-cells and 5 E-cells from 5 different males were tested.

### ORN characterization

In total, six stimuli were used for ORN classification: 10^2^ ng (“low”) and 10^4^ ng (“high”) doses of the pheromone components *E*8-12:Ac and *Z*8-12:Ac; 100 µg of the plant blend (1:1:1 of FAR:MS:TA), and 10 µg of the hair-pencil pheromone, ethyl *trans*-cinnamate. Each contacted sensillum was stimulated with each of the six stimuli in random order (305 cells from 50 female antennae, 45 cells from 12 male antennae).

### Statistical analyses

For EAG we measured the maximum potential generated by the antenna during the 2-s immediately following a puff. For SSR we recorded the number of spikes pre-stimulation (from 2 to 1.5 s before the puff), and the number of spikes post-stimulation (from puff onset to 500 ms). Spike response was estimated as the number of spikes post-stimulation minus the number of spikes pre-stimulation. The reason for not including the spikes immediately before the puff in the pre-stimulation period was that when the puff was loaded with the highest pheromone doses some cells responded as far as 1.2 s before puff onset, probably because the stimulus leaked the pipette, drawn by the continuous air flow (Supplementary Fig. S1 online).

Non-linear regression models were fitted to dose–response curves to estimate the expected half-dose (ED50) (library *drc* in R)^55^. Comparison between a model assuming equal parameters with one assuming different slope, maximum and ED50s were used to determine whether two curves were significantly different from each other^55^. In order to classify cells according to their response to the odorant panel a hierarchical cluster analysis (HCA) based on the maximum distance method was employed (library *Superheat* in R)^56^. Analyses were run in R^57^.

## Supplementary Information

Supplementary Information.

## Data Availability

Raw data and R-script for statistical analysis are available online at https://doi.org/10.34810/data152.
