# Supplementary material for: A closer look at sex pheromone autodetection in the Oriental fruit moth
Source: Sci Rep. 2022 Apr 29;12:7019. doi: 10.1038/s41598-022-10954-x (PMC9055066; doi:10.1038/s41598-022-10954-x)

A closer look at pheromone autodetection in the oriental fruit moth

Pérez-Aparicio, A., Ammagarahalli, B., and Gemeno, C.

Supplementary Material

Contents

Table S1. Number and percentage (mean ± SEM) of sensilla types on the scaled and scale-free areas of one antenna of female *G. molesta*. 2

Table S2. Observed means and SEMs of EAG dose-response curves 2

Table S3. Observed means and SEMs of SSR dose-response curves. 3

Figure S1. Firing activity of the ORNs in the dose-response curves. Each row represents the response of a neuron to a different concentration of either the major (*Z*8-12:Ac) or minor (*E*8-12:Ac) pheromone compounds (left and right, respectively) at one of the 5 concentrations tested (1 ng to 100 µg). Each vertical line represents an individual spike. The two horizontal bars on the top indicate the time of the 200 ms puff. The areas shadowed in pink and green represent sections where spikes were counted to estimate the change in spiking activity before and after the puff, respectively. ORNs where assigned to the *Z*8-12:Ac or *E*8-12:Ac type (right label) based on their relative response to the two isomers (n = 5 cells of each type). 4

Figure S2. Number of sensilla trichodea, sensilla auricillica and sensilla coeloconica along the scaled (SC, n = 5) and scale-free (SF, n = 8) areas of the flageromerers in one antenna of female *G. molesta*. 5

Figure S3. EAG responses of individual antennae of *G. molesta* used in the dose-response curves. 6

Figure S4. SSR responses of the sensilla of *G. molesta* males used to make the dose-response curves. Eleven sensilla were stimulated with 6 doses (1 ng to 100 µg) of the major and minor pheromone components (*Z*8-12:Ac and *E*8-12:Ac, respectively). Six sensilla were classified as major compound specific (Z, right) and 5 as minor compound specific (E, left) based on their relative response to each isomer. 6

Figure S5. Representative SSR traces from male and female *G. molesta* sensilla trichodea responding to female sex pheromone [major (*Z*8-12:Ac) and minor (*E*8-12:Ac) components at 100 ng and 10 µg (Z2, Z4, E2 and E4)], male hair-pencil pheromone (10 µg) and a plant volatile blend (100 µg). The horizontal bar on top of each trace represent the 200 ms puff. 7

Table S1. Number and percentage (mean ± SEM) of sensilla types on the scaled and scale-free areas of one antenna of female *G. molesta*.

Sensillum type	Scaled area (n=8)		Scale-free area (n=5)		Total	
	number	%	number	%	number	%
Trichodeum	466.61 ± 23.32	31.31 ± 00.83	1,215.00 ± 51.56	76.03 ± 00.71	1,681.61	54.44
Auricillicum	768.90 ± 37.36	51.53 ± 01.24	80.40 ± 04.39	05.05 ± 00.31	849.30	27.49
Coeloconicum	157.35 ± 09.36	10.61 ± 00.59	73.80 ± 06.61	04.66 ± 00.49	231.15	7.78
Basiconicum	11.65 ± 06.13	00.72 ± 00.36	101.70 ± 20.20	06.22 ± 00.94	113.35	3.66
Chaeticum	86.22 ± 01.86	05.83 ± 00.21	95.40 ± 03.31	06.03 ± 00.42	181.62	5.87
Styloconicum	00.00 ± 00.00	00.00 ± 00.00	40 ± 2.04	02.00 ± 00.15	31.80	1.02
Total	1490.72 ± 78.03		1598.10 ± 88.27		3,088.82	

Table S2. Observed means and SEMs of EAG dose-response curves

		Females			Males		
**stimulus**	**log10(ng)**	**mean**	**±**	**SEM**	**mean**	**±**	**SEM**
*E*8-12:Ac	0	0.25	±	0.04	0.46	±	0.05
	1	0.25	±	0.04	0.50	±	0.07
	2	0.24	±	0.03	0.51	±	0.05
	3	0.28	±	0.04	0.80	±	0.07
	4	0.72	±	0.09	1.80	±	0.17
	5	1.36	±	0.12	2.30	±	0.21
*Z*8-12:Ac	0	0.23	±	0.03	0.45	±	0.06
	1	0.21	±	0.03	0.57	±	0.07
	2	0.25	±	0.03	1.00	±	0.11
	3	0.35	±	0.04	1.71	±	0.14
	4	0.91	±	0.11	2.82	±	0.26
	5	1.52	±	0.12	2.66	±	0.29

Table S3. Observed means and SEMs of SSR dose-response curves.

ORN type	stimulus	log10(ng)	mean		SEM
E	E8-12:Ac	0	1.40	±	1.14
		1	2.20	±	1.59
		2	15.40	±	3.01
		3	33.20	±	2.44
		4	39.40	±	3.90
		5	40.20	±	6.71
	Z8-12:Ac	0	3.20	±	0.80
		1	2.60	±	0.76
		2	4.40	±	1.53
		3	10.60	±	2.67
		4	25.10	±	3.53
		5	33.30	±	3.89
Z	E8-12:Ac	0	-0.58	±	0.93
		1	-0.67	±	0.40
		2	1.33	±	0.85
		3	2.00	±	1.26
		4	25.67	±	4.20
		5	35.92	±	3.10
	Z8-12:Ac	0	1.50	±	0.71
		1	3.50	±	1.30
		2	14.33	±	2.71
		3	30.92	±	3.75
		4	36.33	±	3.41
		5	33.58	±	4.75

Figure S1. Firing activity of the ORNs in the dose-response curves. Each row represents the response of a neuron to a different concentration of either the major (*Z*8-12:Ac) or minor (*E*8-12:Ac) pheromone compounds (left and right, respectively) at one of the 5 concentrations tested (1 ng to 100 μg). Each vertical line represents an individual spike. The two horizontal bars on the top indicate the time of the 200 ms puff. The areas shadowed in pink and green represent sections where spikes were counted to estimate the change in spiking activity before and after the puff, respectively. ORNs where assigned to the *Z*8-12:Ac or *E*8-12:Ac type (right label) based on their relative response to the two isomers (n = 5 cells of each type).

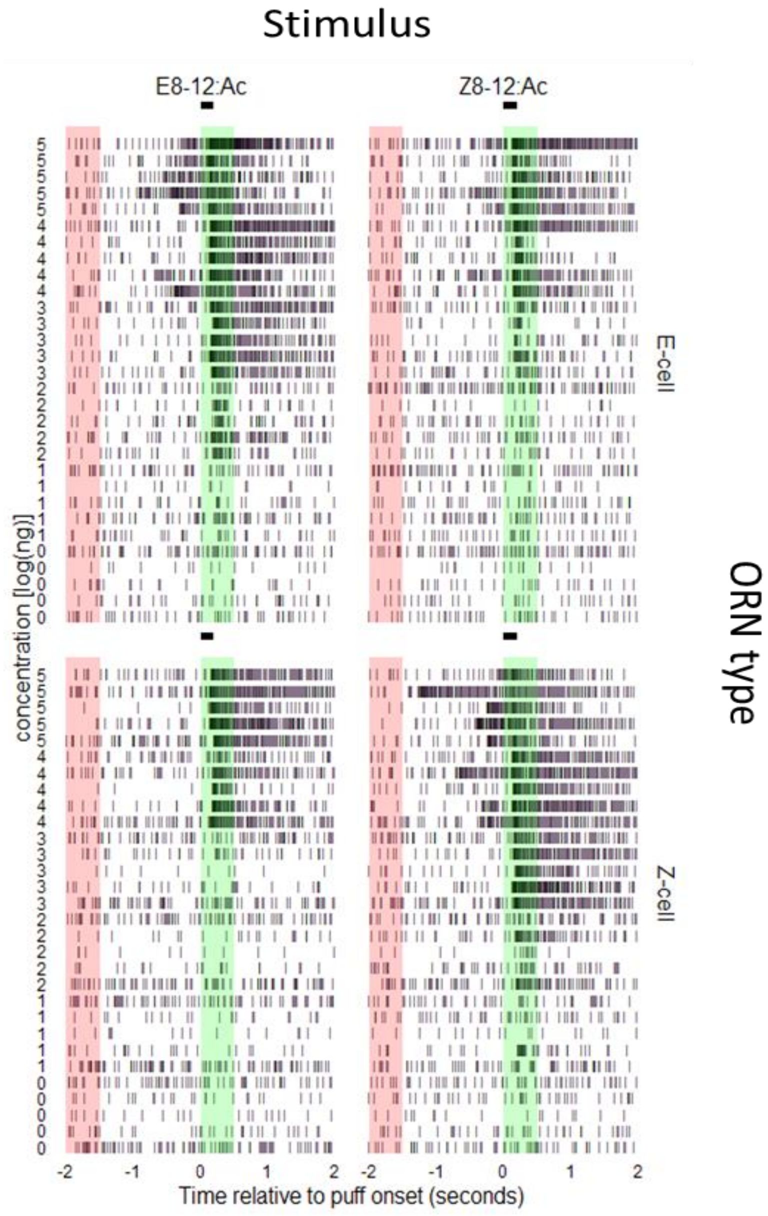

Figure S2. Number of sensilla trichodea, sensilla auricillica and sensilla coeloconica along the scaled (SC, n = 5) and scale-free (SF, n = 8) areas of the flageromerers in one antenna of female *G. molesta*.

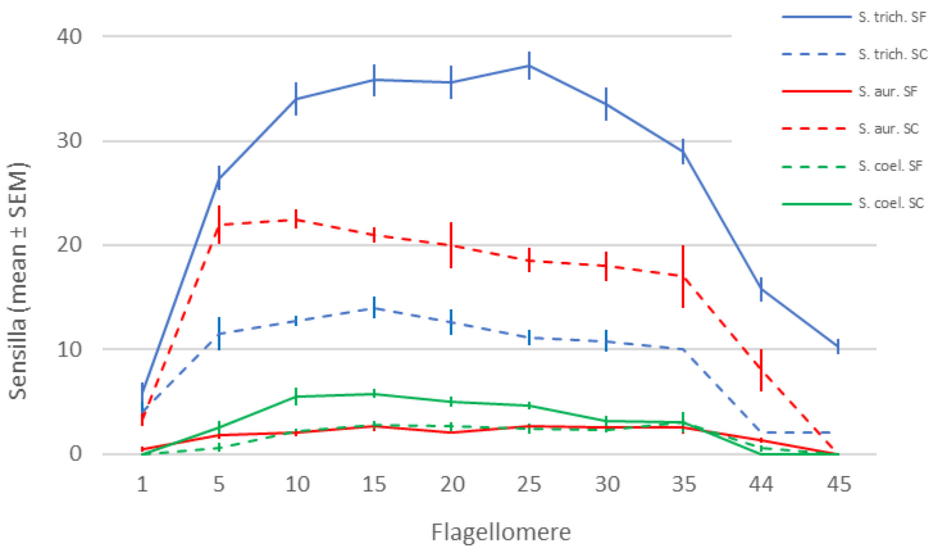

Figure S3. EAG responses of individual antennae of *G. molesta* used in the dose-response curves.

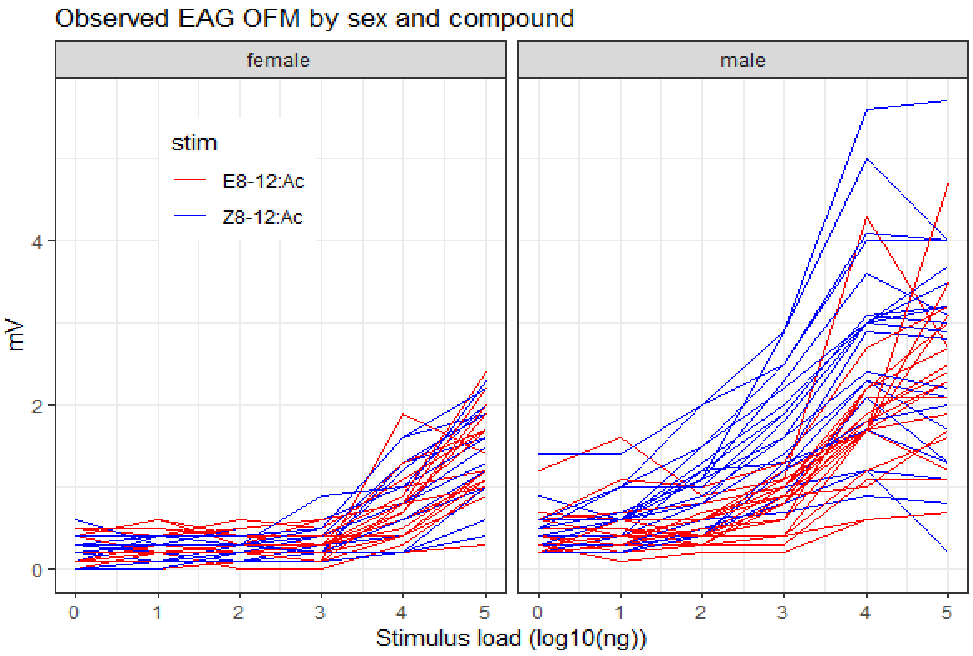

Figure S4. SSR responses of the sensilla of *G. molesta* males used to make the dose-response curves. Eleven sensilla were stimulated with 6 doses (1 ng to 100 μg) of the major and minor pheromone components (*Z*8-12:Ac and *E*8-12:Ac, respectively). Six sensilla were classified as major compound specific (Z, right) and 5 as minor compound specific (E, left) based on their relative response to each isomer.

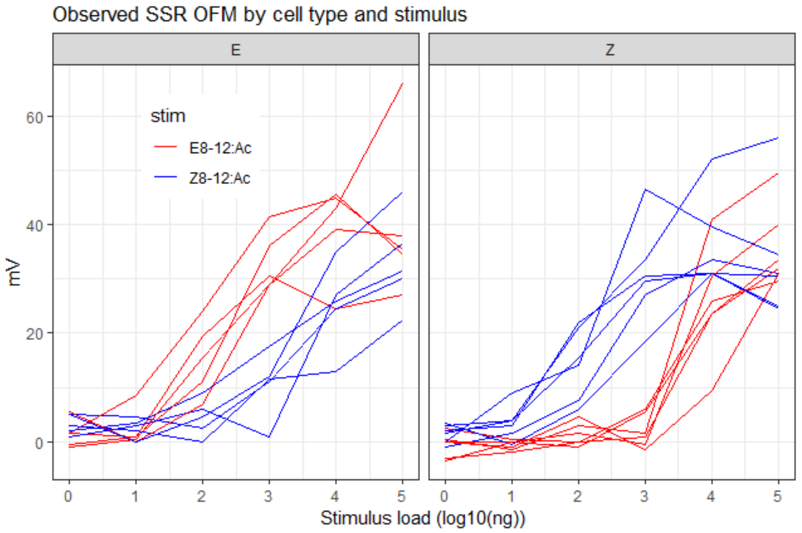

Figure S5. Representative SSR traces from male and female *G. molesta* sensilla trichodea responding to female sex pheromone [major (*Z*8-12:Ac) and minor (*E*8-12:Ac) components at 100 ng and 10 μg (Z2, Z4, E2 and E4)], male hair-pencil pheromone (10 μg) and a plant volatile blend (100 μg). The horizontal bar on top of each trace represent the 200 ms puff.

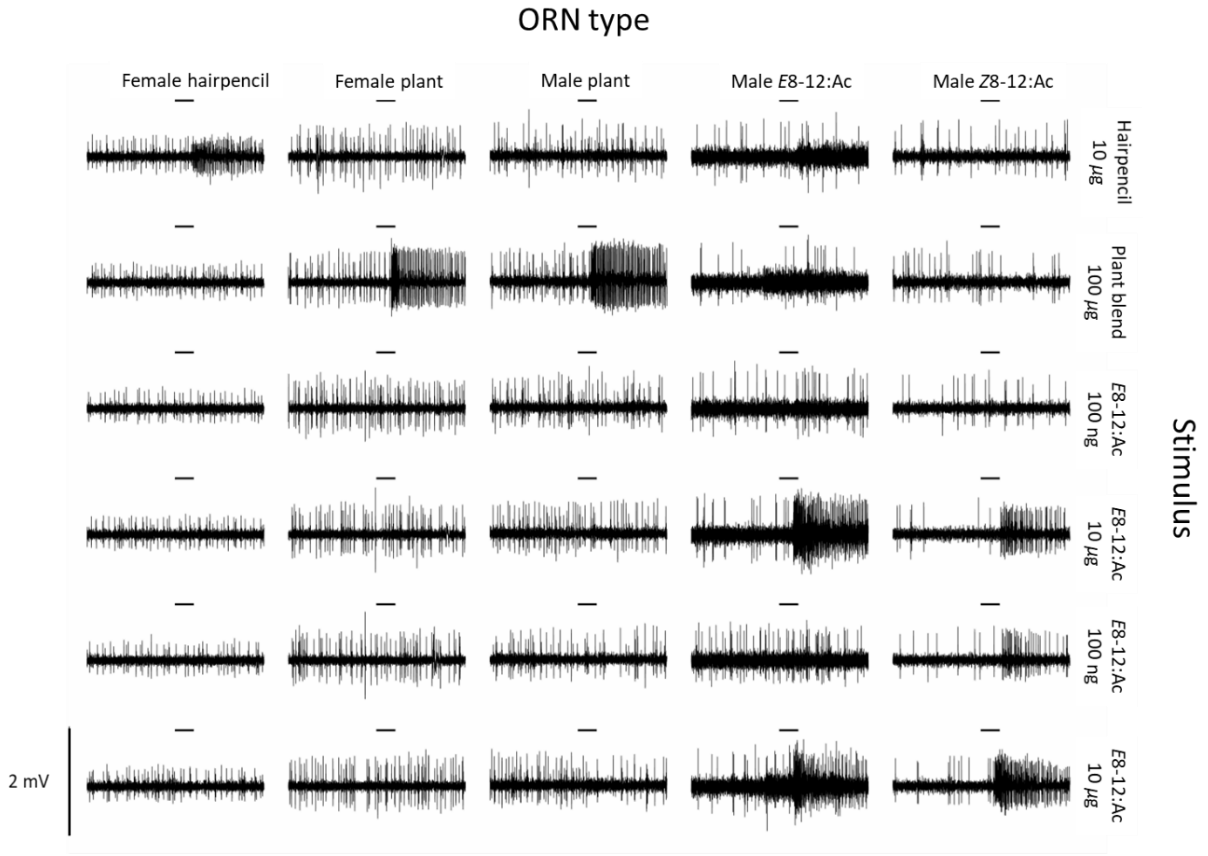

Supplement: Supplementary file 1 — Supplementary Information. [file 41598_2022_10954_MOESM1_ESM.pdf]
